# The Spread of *Aedes albopictus* in Metropolitan France: Contribution of Environmental Drivers and Human Activities and Predictions for a Near Future

**DOI:** 10.1371/journal.pone.0125600

**Published:** 2015-05-11

**Authors:** Benjamin Roche, Lucas Léger, Grégory L’Ambert, Guillaume Lacour, Rémi Foussadier, Gilles Besnard, Hélène Barré-Cardi, Frédéric Simard, Didier Fontenille

**Affiliations:** 1 UMI IRD/UPMC UMMISCO, Bondy, France; 2 UMR CNRS/IRD/UM1/UM2 MIVEGEC, Montpellier, France; 3 EID Méditerranée, Montpellier, France; 4 Centre de Recherche sur la Biodiversité, Louvain-la-Neuve, Belgique; 5 EID Rhône-Alpes, Chindrieux, France; 6 Office de l’Environnement de la Corse, Corte, France; Universidade Federal do Rio de Janeiro, BRAZIL

## Abstract

Invasion of new territories by insect vector species that can transmit pathogens is one of the most important threats for human health. The spread of the mosquito *Aedes albopictus* in Europe is emblematic, because of its major role in the emergence and transmission of arboviruses such as dengue or chikungunya. Here, we modeled the spread of this mosquito species in France through a statistical framework taking advantage of a long-term surveillance dataset going back to the first observation of *Ae*. *albopictus* in the Metropolitan area. After validating the model, we show that human activities are especially important for mosquito dispersion while land use is a major factor for mosquito establishment. More importantly, we show that *Ae*. *albopictus* invasion is accelerating through time in this area, resulting in a geographic range extending further and further year after year. We also show that sporadic “jump” of *Ae*. *albopictus* in a new location far from the colonized area did not succeed in starting a new invasion front so far. Finally, we discuss on a potential adaptation to cooler climate and the risk of invasion into Northern latitudes.

## Introduction

Today, biological invasions are especially studied in order to overcome their negative impact for biodiversity conservation [1]. However, some invasive species can also raise serious public health issues. This is the case of some insect species, especially mosquitoes, that can transmit numerous pathogens [2]. Indeed, several factors such as potentially climate change [3,4] and tire transportation [5] have significantly contributed expanding their geographic distribution and thus increasing the human population at risk of transmission.

Global invasion of *Aedes albopictus* is especially important to understand in this context [6]. This mosquito species is an aggressive day-biting insect, that is able to transmit at least dengue and chikungunya viruses [7–10]. The 2002 epidemic in the USA has also suggested that this mosquito can be an important bridge vector for West Nile virus [11]. Its geographic expansion raises serious public health concerns, especially in Western Europe because of repeated introduction of dengue and chikungunya viruses through imported cases [12]. Such importations have already initiated local transmission of dengue viruses in France [13,14] and Croatia [15] even leading to a chikungunya outbreak in Italy [16].

In South-East Asia, where the species is native, *Ae*. *albopictus* was originally considered a rural mosquito [17] because it preferentially bred in natural habitats and was mainly present at the edge of forests. However, the species is now widespread in suburban and urban environments where its larvae thrive in man-made water containers (water storage containers, abandoned tins, tires…). At the adult stage, although *Ae*. *albopictus* females are able to obtain blood from a wide range of hosts depending on their availability and the environment [18,19], humans are clearly preferred when available [7,18]. The presence of this highly competent vector in dense human ecosystems combined with its highly anthropophilic behavior amplifies its potential negative effect for public health.

*Aedes albopictus* colonization of Europe started four decades ago [20]. Introduction was first documented in Albania during the late 1970s, followed by Italy in the 1990s, from where it gradually spread into other Mediterranean countries, including France, Spain, Slovenia, Croatia, Bosnia and Herzegovina, Montenegro, and Greece [20]. Maritime transportation of tires and other goods have been suspected to play a large role in supporting the dispersal of *Ae*. *albopictus* across continents [5] and some environmental changes probably aided establishment and further spread [21]. Today, this mosquito has colonized every continents, except Antarctica, and expansion northwards is clearly expected [20,21].

Several studies have already statistically addressed environmental drivers of *Ae*. *albopictus* invasion. At a very large scale, a recent study by the European Center for Diseases Control (2013) [22] has shown that mosquito presence is mainly driven by rainfall and day time surface temperature. According to this model, *Ae*. *albopictus* is expected throughout most of the French territory, including also Northern areas. Following a similar approach, several studies have highlighted that climate change is expected to increase significantly the spatial distribution of this species, especially when the evolutionary dimension is considered [23]. To this extent, the European continent seems especially at risk [3,24]. However, the role of human transportation has not been assessed so far in these statistical models.

In this study, we aimed at characterizing the current process of *Ae*. *albopictus* spread in Metropolitan France. We developed a statistical model to estimate the contribution of different parameters potentially involved in this process and we show that human transportation is key to the geographic range expansion of this mosquito, and that environmental factors such as land use determines establishment. We also show that the colonization process is currently accelerating. We finally discuss the most likely dispersal routes for *Ae*. *albopictus* in the near future.

## Materials and Methods

### 2.1. Context of Aedes albopictus invasion in metropolitan France

Invasion is a complex process [25], especially difficult to model [26] because it is shaped by many factors, such as local diversity of other species [27], that also evolves through time [28]. Nevertheless, spatio-temporal dynamics of invasive species do not seem to be idiosyncratic and indeed follow some general rules across different taxa [29]. A very common pattern in biological invasion is a neighborhood diffusion combined with sporadic jumps [26]. To this extent, *Ae*. *albopictus* seems to follow this kind of general pattern since the invasion wave in Metropolitan France is progressive and gradual since its introduction in Menton in 2004 [30]. However, some locations, especially on resting areas along highways, show positivity despite standing far apart from the colonization front, suggesting the occurrence of sporadic “jumps”. Since this mosquito is highly anthropophilic [18], we will assume that human transportation is a key factor for *Ae*. *albopictus* dispersal [31].

### 2.2. Data

#### 2.2.1. Entomological data

**Fig 1:**
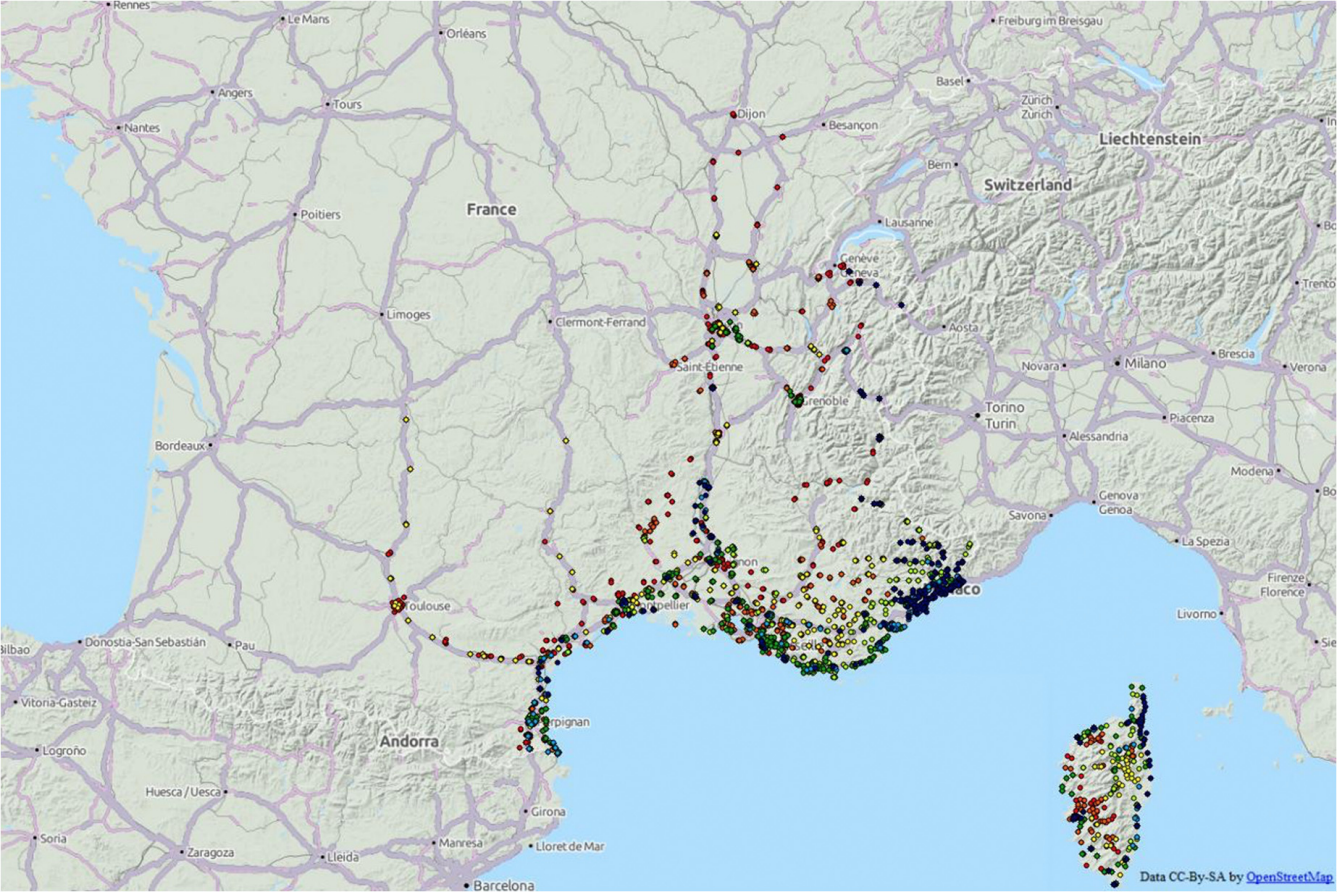
Location and collection year of traps. Green, yellow, blue, red, orange are the traps collected in 2006, 2007, 2008, 2009, 2010 respectively.

Our study capitalized on routine surveillance data based on mosquito oviposition traps (*i*.*e*. ovitraps) monitoring between 2006 and 2012 in Southern France (Fig 1) operated by the Entente Interdépartementale de Démoustication (EID). Each ovitrap was sampled monthly between June and November each year, and can be positive or negative (presence or absence of *Ae*. *albopictus* eggs or larvae). In order to be analyzed through a statistical framework, entomological data were aggregated into two time lags each year, *i*.*e*., before and after July 31^st^. We thus consider two time steps. During the first semester, *Ae*. *albopictus* populations consist of diapauses survivors and effective population increases slowly, corresponding to the *implantation period* (*i*.*e*., population growth). From August, adult populations are assumed to have emerged and then can be passively dispersed, corresponding to the *dispersion period* (*i*.*e*., geographical spread). For each trap, a semester is considered positive if it has been observed in a positive state during at least 3 consecutive months. We have characterized our data at a semester scale in order to take into account only real presence. Indeed, some traps can reveal sporadic presence during one month, which should not be considered as a local implementation of the mosquito, but rather as an accidental dispersal with no consequences on the whole invasion dynamics of the species.

For logistic reasons, mosquito traps have been displaced through years when locations were invaded with high certainty. Thus, for each location, if two consecutive semesters are positive and the location is not recorded anymore after these two semesters, the geographic location is considered positive for all subsequent time points, *i*.*e*., the mosquito population is considered established. In turn, if a new trap is negative for two consecutive semesters, the location is assumed to be negative before the beginning of data collection.

#### 2.2.2. Environmental and human variables

Proliferation of *Ae*. *albopictus* requires some abiotic conditions that has been analyzed elsewhere [32]. In order to include these abiotic factors into our analysis, we have used freely available datasets estimate the contribution of several environmental variables to *Ae*. *albopictus* dispersal, including:

*Land-use*. Land-cover types have been characterized using the Corine Land Cover database from 2006 (resolution 100m, http://www.eea.europa.eu/data-and-maps/data/corine-land-cover-2006-raster-2). 46 different land-cover types are included in Corine Land Cover version 16 [33] that have categorized within 6 levels (urban, peri-urban, transportation area, urban park, agricultural landscape and forest) and therefore included within this analysis.

*Climatic data*. Climatic data have been downloaded from the website http://www.worldclim.org/ using a 30-arc seconds resolution. Variables downloaded included: annual minimum temperature, annual maximum temperature, annual mean temperature, annual precipitation, altitude, maximal temperature of warmest month, minimal temperature of coldest month and temperature seasonality.

*Distance to the colonized area*. To account for human-assisted migration, distance was defined as traveling time, *i*.*e*., weighted by the average traffic speed on a given land-use. Here, we assumed that traffic speed rates are 130km/h on highways, 80 km/h on national roads, 50km/h for rural roads (corresponding to the French speed limits on the different roads) and 1km/h for non–practicable areas such as forests. This distance has been calculated using the OpenStreetMap database (http://download.geofabrik.de/europe/france/) and has been squared-root transformed in order to avoid an exaggeratedly long distribution tail.

### 2.3. Methods

#### 2.3.1. Estimation of *Ae*. *albopictus* dispersal (introduction of the invasive species)

We first used a logistic model to estimate *Ae*. *albopictus* dispersal without any constraint. This logistic model was fitted first with positive trap observations during the first semester of 2006 and distance from Menton (43.77N and 7.49W) and Bastia (42.69N and 9.45W), the two starting points of *Ae*. *albopictus* in continental France and Corsica island, respectively. Then, we considered that a probability of presence of *Ae*. *albopictus* immature stages in a given trap greater than 50% (corroborated by additional analyses, see appendix 2) means that the geographic location of the trap is colonized. Thus, we estimated the probability of a trap being positive during the 2^nd^ semester of 2006 according to the distance to the colonization front in the 1^st^ semester (*i*.*e*. the closest trap colonized), rather than distance from locations in the core of the previously colonized area (*i*.*e*., Menton and Bastia). This iterative process was repeated until 2^nd^ semester of 2012.

We also considered a second distance when the newly colonized area is not adjacent to the previous one, *i*.*e*., when a “jump” (or long-distance dispersal) has occurred. Indeed, the invasion of this species clearly follows a “wave” pattern from the Italian border. Despite this wave represents the main invasion front (what we call “colonized area”), some “jumps” from this area, *i*.*e*., efficient long distance dispersal with a local presence for at least three consecutive months, can nevertheless occur. Then, a “jump” is defined as a positive trap outside of the colonized area. Then, distance from the closest such propagule has also been considered in the logistic model. No distance limitation where considered to consider such “jump”. Distance to colonized area and distance to a jump from colonized areas were considered in the analysis. Results from univariate models with these distances are presented in the supplementary materials (Tables B–K in S1 File).

#### 2.3.2. Estimation of environmental factors for successful invasion of *Ae*. *Albopictus* (establishment)

In a second step, a generalized-mixed model was used to explain actual differences between the colonized area estimated by the previous logistic simple model and the presence/absence of each trap recorded (*i*.*e*., the residuals of the logistic model). The model includes all environmental variables as fixed effect and trap location as a random effect. Variables present in the final model have been selected according to their significant contribution or not, following an iterative stepwise process with a forward approach: each variable was tested alone and the most significant was kept, then each remaining variable was tested in addition to the previously selected one(s) and the combination with the best AIC was kept.

#### 2.3.3 Validity of the model

Robustness of our model has been estimated by three different ways. First, we have quantified the area under the curve between outputs of statistical model and observed data. Second, we made geographic buffer of 1 km^2^ throughout the territory around each trap and compared the probability of positive traps generated by our model with the proportion of *Ae*. *albopictus* positive traps observed. Finally, we quantify the validity of our model by comparing, for each semester, the number of positive and negative traps recorded in areas where our model predicts a probability of observation greater and lower than 50% respectively.

#### 2.3.4 Prediction

To predict the spread of *Ae*. *albopictus*, we used the same “two-steps” approach as described above. First, we applied the logistic model to estimate the new area that should be colonized in semester N+1 from trap data obtained in semester N. Then, we applied the coefficients of the generalized mixed model to estimate the new geographic range of *Ae*. *albopictus* with environmental constraints. The coefficients of the model used for predictive outcome are the averaged coefficients at the semester level over the last three years in order to avoid effects due to the increasing surveillance effort tracking species invasion. All analyses have been done with ArcGis v10.2.

## Results

The methodology described above was applied to the entomological dataset recorded between 2006 and 2011 from Southern France and Corsica. Model fitting was satisfying since the Area Under Curve (AUC) was estimated at 0.98 (Table 1). The relationship between predicted probability of observation and proportion of positive traps observed in the same area shows a slope close to 1 with small variability (section A in S1 File). Finally, 95% of locations are correctly classified when locations are assumed positive or negative with an observation probability greater or lower than 50% (section B in S1 File).

**Table 1:** Results of statistical modeling.

Variable	Coefficient	Odds Ratio
**Agricultural landscape**	**0.725 (0.052;1.398)**	**2.065**
**Peri-urban landscape**	**0.756 (0.142;1.370)**	**2.130**
**Urban landscape**	**1.189 (0.572;1.805)**	**3.284**
**Second semester**	**1.797 (1.559;2.035)**	**6.035**
**Minimum temperature of the coldest month**	**0.069 (0.055;0.082)**	**1.071**
Distance to colonized area*colonized area	**12.804 (11.657;13.951)**	**3.638e+05**
Second semester*minimum temperature of the coldest month	**-0.021 (-0.028;-0.014)**	**0.978**
Distance to colonized area*year	**2.628 (2.292;2.964)**	**13.85**
**Distance to colonized area at previous semester**	**-8.868 (-9.453;-8.282)**	**1.408e-04**
**Distance to area sporadically colonized**	**-6.146 (-7.272;-5.021)**	**2.140e-03**
**Area Under the Curve (AUC):**	**Fixed effect: 0,96**	
	**Fixed and random effect: 0,99**	
	**R2c = 0.883**	

All variables have a significant effect of the probability to observe *Aedes albopictus*. The interaction between the distance to colonized area and colonized area positive shows that probability of observing mosquito presence is higher in the middle of the colonized area than on its edges.

Furthermore, Table 1 shows that land use is an important predictor for mosquito presence. Peri-urban and urban areas appear as the most suitable areas for *Ae*. *albopictus* establishment, especially urban areas where the probability to observe *Ae*. *albopictus* is 65% greater than peri-urban areas.

The major impact of man-assisted transportation is apparent since presence probability decreases with distance, in travelling time, to the colonized area. Moreover, the significant interaction between distance to the colonized area and the year of establishment underlines that the slope of the relationship between these two quantities decreases through time. In other words, the yearly pace of *Ae*. *albopictus* spread in continental France is increasing year after year, suggesting an acceleration of the invasion process.

**Fig 2:**
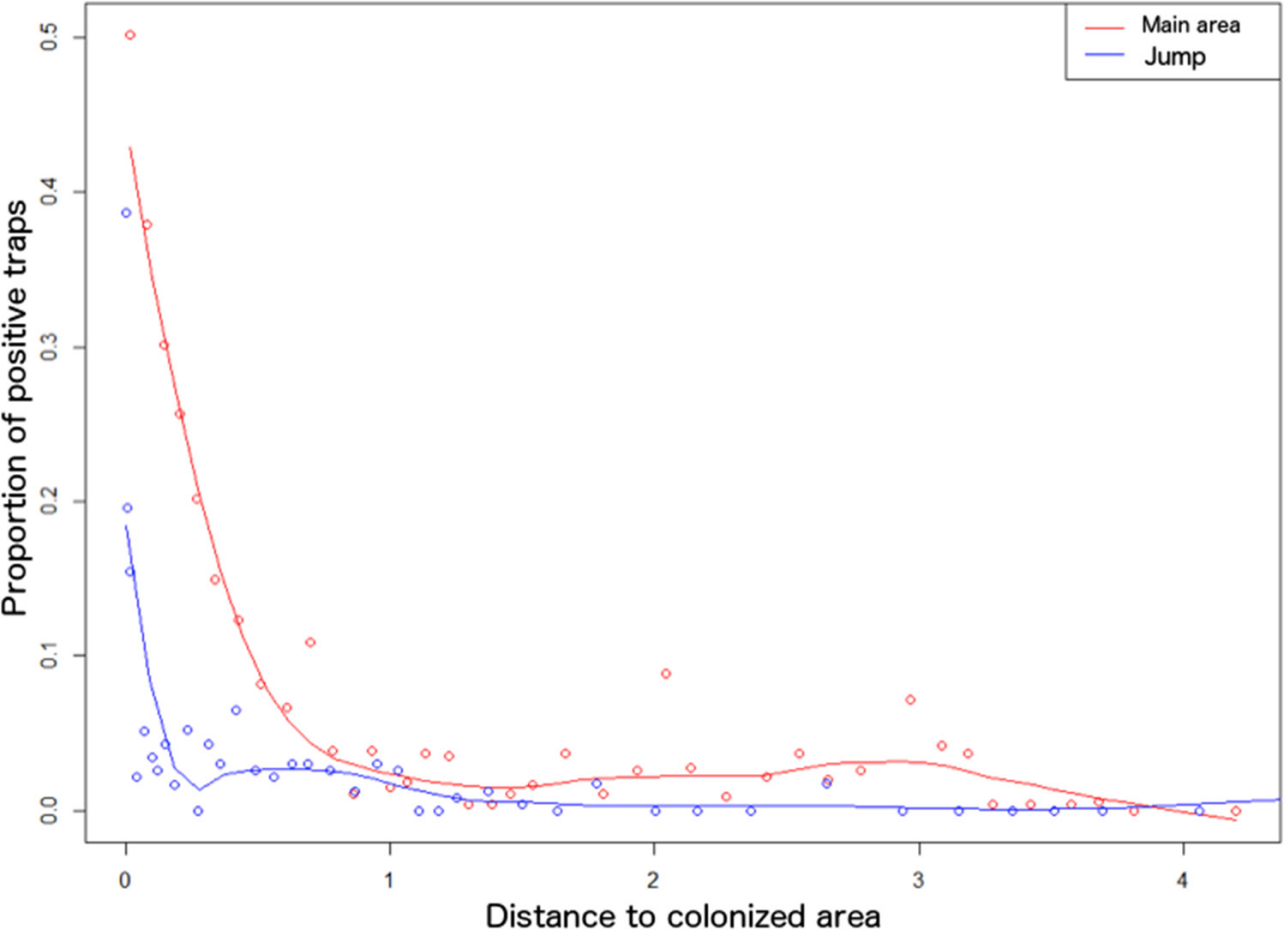
Relationship between proportion of positive traps and distance to main colonized area (red line) and to area colonized sporadically (“jump”, blue line). The x-axis is in km. The gradual relationship shown on the red line suggests an invasive wave. Conversely, abrupt drop of the blue line suggests that such “jump” did not result so far in a new front of invasion. Curves have been fitted through loess algorithm.

The relation between the distance to colonized area and *Ae*. *albopictus* presence is different if we consider the “main colonized area” or the “sporadic jumps” of this mosquito that occur every year (Fig 2). Indeed, the proportion of positive traps decreases gradually with distance to the main colonization area, suggesting that invasion is following neighborhood dispersal as previously suggested. Conversely, the proportion of positive traps decreases abruptly with distance to areas that are sporadically colonized. This pattern suggests that most of the propagules resulting from long-distance “jumps” have not started new colonization fronts so far.

## Prediction

We used averaged values, for each semester, of coefficients estimated over the last three years to predict the probability of occurrence of *Ae*. *albopictus* in new areas in 2013 and 2014. Consequently, the probability of location positivity for the first semester was obtained through:

Logit(Positivity)=1.8387−3.9972*sqrt(distancetocolonizedarea)−6.4300*sqrt(distancetoareasporadicallycolonized)+11.6729*sqrt(distancetocolonizedarea)*(Colonizedarea)

**Fig 3:**
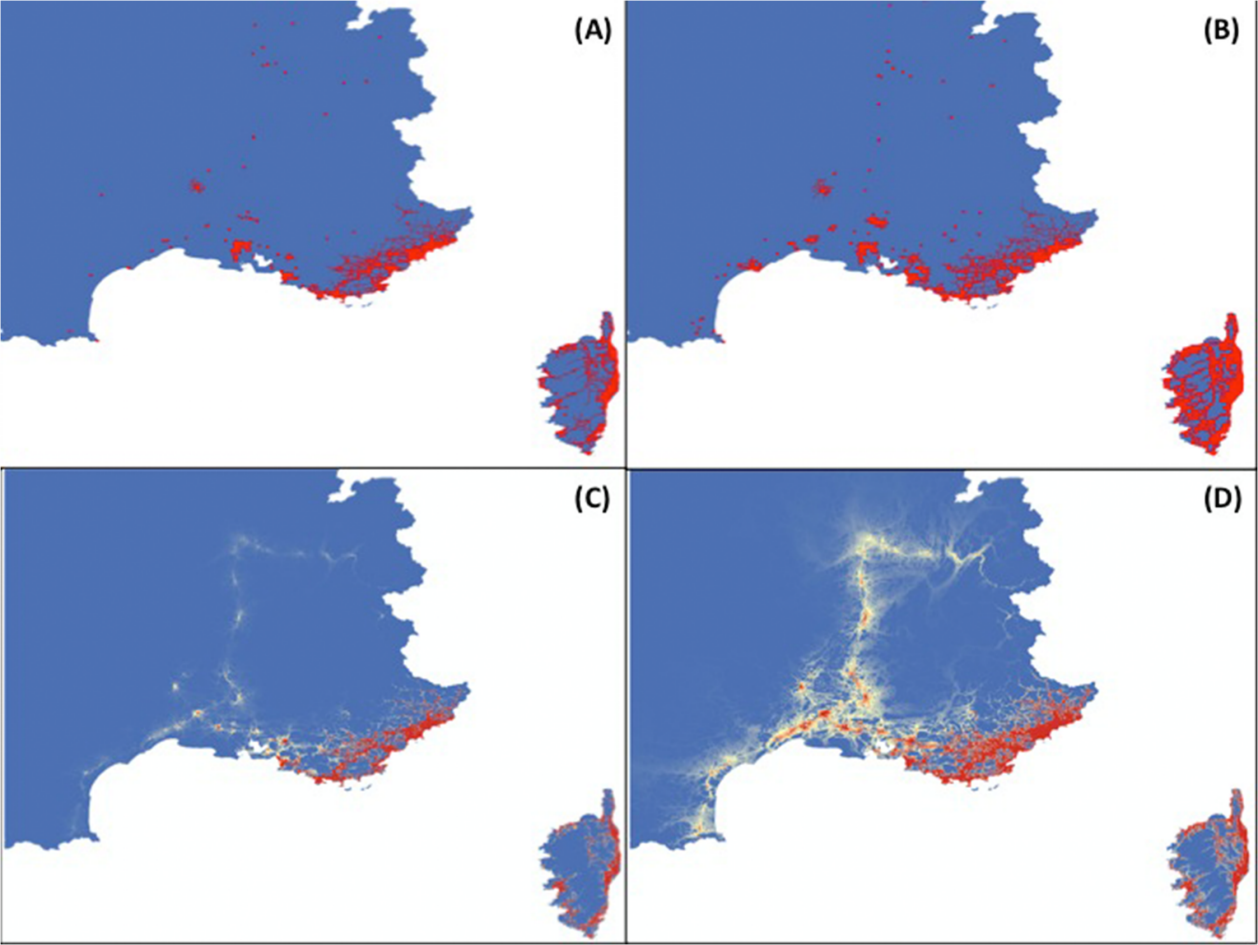
Positive traps observed (A,B) and model predictions (C,D) for 2012. (A,C) First semester; (B,D) Second semester. Values range from probability of 0% to observe *Ae*. *albopictus* (blue) to 100% (red).

**Fig 4:**
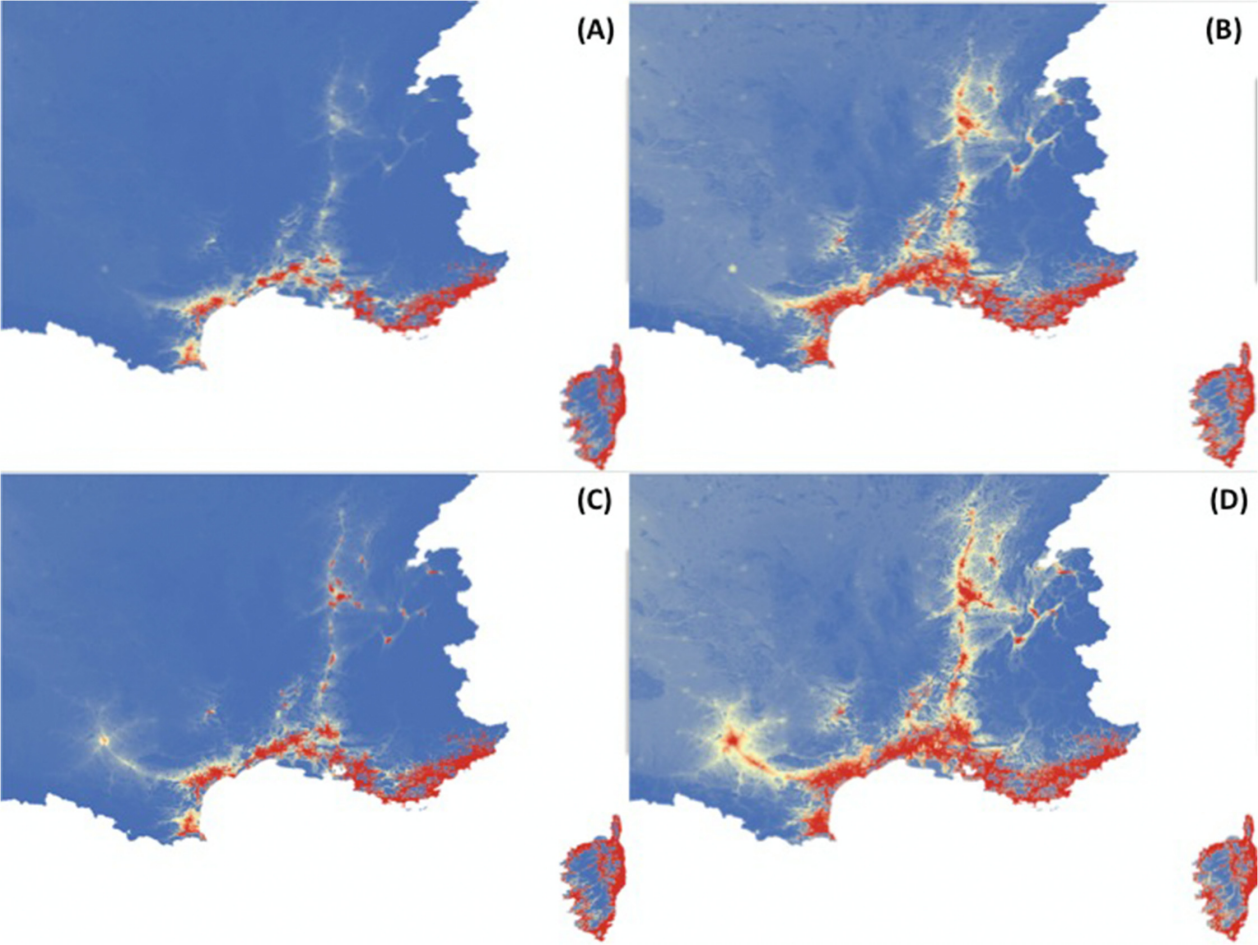
Prediction of probability of presence of *Aedes albopictus* in 2013 (A,B) and 2014 (C,D) for the first and second semesters (A,C and B,D respectively). Values range from probability of 0% to observe *Ae*. *albopictus* (blue) to 100% (red).

And, for the second semester:

Logit(Positivity)=4.9795−4.5312*sqrt(distancetocolonizedarea)−11.820*sqrt(distancetoareasporadicallycolonized)+10.8559*sqrt(distancetocolonizedarea)*(Colonizedarea)

which represents the most parsimonious model allowing having predictions for 2012 in good agreement with observations (Fig 3). Our model predicts, for 2013 and 2014, that *Ae*. *albopictus* will extend its distribution area northwards and westwards, following mostly highways present in this area (Fig 4). These predictive maps will be produced on a regular basis and will be posted on the website of “Centre National d’Expertise des Vecteurs” (CNEV, http://www.cnev.fr).

## Discussion and Conclusions

In this study, we have described the spread of *Ae*. *albopictus* in continental France and Corsica and quantified the main drivers of its invasion dynamics. We found that land use is fundamental for the establishment of new mosquito propagules, and that urban and peri-urban areas were particularly suitable areas. We also showed that *Ae*. *albopictus* is currently surfing a dispersal wave, with occasional “jumps” that did not yet result in new colonization fronts. However, the mosquito colonizes locations that are further and further apart each year, a clear signal of an acceleration of this dispersal process. Finally, we show that the invasion wave is growing during the second semester, validating our assumption of a two-step process where dispersal is mainly operated through adults. The good agreement between entomological data collected in 2012 and our predictions for this period with our model trained on data until 2011 allows us to make predictions about future routes of dispersal, highlighting a major risk for establishment of *Ae*. *albopictus* in highly urbanized areas along the dense highways network towards the North and West parts of France.

The statistical model presented here clearly depends on the quality of the data. Since our dataset is collected for public health outcomes, spatial sampling is not uniform and constant through time, which can introduce a bias in our estimates. However, more than 1000 traps (1316 during the second semester of 2012) have been used during the last 6 years. Thus, this dataset is probably one of the most robust available to date to explore this invasion event and we are confident that the high number of traps should overcome the potential bias in spatial sampling.

Three aspects of our approach have been chosen arbitrarily and deserve discussion. First, we use predefined speed rates to calculate the distance to the colonized areas. Although we considered regular speed used in France, another possibility would have been to randomize values for speed. Nevertheless, calculating distance at such scale (Southeast of France) with the high resolution required to study mosquito invasion (pixel are 100m x 100m) is too computing intensive to address a large range of other values. The other aspect that has been the result of a modeling choice is the number of years averaged to make prediction. However, values of model coefficients tend to stabilize through time, then including a different period of time would not have a profound impact. Finally, we have assumed that a trap location cannot “recover” when *Ae*. *albopictus* has colonized it. Even if this assumption could be strong, it is consistent with current knowledge on *Ae*. *albopictus* in South of France where no disappearance of this mosquito has been recorded so far.

As for any biological invasion, it is known that the invader changes through time [28]. This is the reason why a lag is often observed between the arrival of an invader and its explosive population growth because invaders generally need specific adaptations [34] or acclimations [35], which is also consistent with the acceleration of dispersal observed in our study. Another explanation for this lag could be the time required to obtain a sufficient population size for an efficient dissemination; indeed, about 5 years were necessary for *Ae*. *albopictus* population to reach stable population size in Nice [36].

Our study suggests that some climatic factors are especially important for the presence of *Ae*. *albopictus*, such as minimal temperature of the coldest month, which remains consistent with previous modeling studies. However, other climatic factors, such as rainfall, do not appear to play a major role within our modeling framework, despite its role has been previously highlighted in other studies (ECDC, 2013). This difference could be explained by the fact that our study focuses on *Ae*. *albopictus* in metropolitan France, with a spatial distribution is limited to Southern France where rainfall patterns show low variability. It is also possible that some climatic factors have a quantitative effect on mosquito abundance, but not on its presence. In addition of the previous studies, our work reveals, thanks to the high-resolution level available with our dataset, the significant role of human transportation in the invasion dynamics of *Ae*. *albopictus*. Land-use is also especially important, with a greater risk to observe the mosquito in urban areas, which is also consistent with current literature [20]. Since our study suggests that invasion dynamics is currently accelerating throughout the French territory, it was especially important to figure out the drivers of dispersion in order to anticipate next steps of the invasion wave.

*Aedes albopictus* has demonstrated ability to adapt to seasonal variations through photoperiodic diapause [35,37]. During short day-length in temperate climates, adult females of *Ae*. *albopictus* oviposit eggs in which pharate larvae enter diapause inside the chorion of the egg until permissive conditions favor the resumption of development [37]. This mechanism is mainly regulated by the critical photoperiod (seasonal timing of diapause) and the diapause incidence. As shown in invasive populations of *Ae*. *albopictus* from the USA [38], rapid evolution in these two traits is a crucial element of the invasion potential [37,38]. It would then be particularly important to study *Ae*. *albopictus* in the Northern part of the colonized area to figure out if evolution has already taken place. Moreover, studying the adaptive velocity of these traits in *Ae*. *albopictus* in the Northern part of the colonized area would be particularly informative to figure out the possible spread speed toward north latitudes. Thus, this model will have to be updated when the species will reach northern latitudes in order to re-evaluate the contribution of climatic factors and thus to keep its predictive ability.

Furthermore, it is well-known that temperature strongly influences *Ae*. *albopictus* growth, emergence and mortality rates [32]. A difference in 4°C is enough to see a mosquito density divided by two [32], which is definitely the case between French sites of introduction (at Italian borders) and northernmost areas of colonization (in center of France). However, proximal mechanisms allowing *Ae*. *albopictus* to express better resistance to cold temperatures remain to be discovered. Indeed, a natural next step is to wonder when *Ae*. *albopictus* will stop its invasion towards Northern France, which exhibits significantly cooler climates. However, the ending point of this invasion cannot be determined without figuring out the evolutionary trade-offs in action for these life-history traits.

Once *Ae*. *albopictus* is established in an area it is excessively difficult to eliminate, and constant surveillance and appropriate control strategies are required [39]. Indeed, among the main control strategies that can be applied against *Ae*. *albopictus* [40], none of them seems to be efficient in the field. However, the number of imported cases of dengue viruses or chikungunya in Western Europe is pretty high [12,13]. Since the spread of *Ae*. *albopictus* seems unavoidable, it calls for urgent implementation of epidemiological modeling [41,42] within this kind of ecological model in order to predict what could be, year after year, the most exposed areas to local outbreaks.

This model is of particular importance for public health authorities. Indeed, such tools, able to predict invasion of a mosquito that can serve as a potential vector of many pathogens, allows improving surveillance and risk forecasting through a better and more representative spatial sampling. Implementation of such a tool for vector control management will be critical, especially in areas where public health authorities are not used to controlling mosquito and/or in large population basins where multiple imported cases could rapidly result in local virus transmission.

## Supporting Information

S1 File Fig A, Relationship between predicted and observed data of *Ae*. *albopictus* presence. Fig B, Distribution of *Ae*. *albopictus* presence probability within negative traps (in blue) and positive traps (in red). Table A, Correlations between variables included within the predictive model. No correlation is significant. Tables B1 and B2, Univariate models with distance to a jump from the colonized area (with a random effect ‘trap’). Table C, Probability of positive trap according to location within the colonized area. Trap is included as a random factor. Table D, Probability of positive trap according to the interaction between the location in colonized area (yes/no) and the distance to this colonized area. Trap is included as a random factor. Table E, Probability of positive trap according to the distance to colonized area and the interaction between the location in colonized area (yes/no) and the distance to this colonized area. Trap is included as a random factor. Table F, Probability of positive trap according to the type of landscape. Trap is included as a random factor. Table G, Probability of positive trap according to the current year. Trap is included as a random factor. Table H, Probability of positive trap according to the interaction between the current year and the distance to colonized area. Trap is included as a random factor. Table I, Probability of positive trap according to the mosquito presence during second semester. Trap is included as a random factor. Table J, Probability of positive trap according to the minimal temperature. Trap is included as a random factor. Table K, Probability of positive trap according to the minimal temperature, presence during second semester and their interaction. Trap is included as a random factor. Table L, Full model with trap and semester as random factor.
(DOCX)
