# Supplementary material for: The Spread of Aedes albopictus in Metropolitan France: Contribution of Environmental Drivers and Human Activities and Predictions for a Near Future
Source: PLoS One. 2015 May 11;10(5):e0125600. doi: 10.1371/journal.pone.0125600 (PMC4427491; doi:10.1371/journal.pone.0125600)
Supplement: S1 File — Fig B, Distribution of Ae. albopictus presence probability within negative traps (in blue) and positive traps (in red). Table A, Correlations between variables included within the predictive model. No correlation is significant. Tables B1 and B2, Univariate models with distance to a jump from the colonized area (with a random effect ‘trap’). Table C, Probability of positive trap according to location within the colonized area. Trap is included as a random factor. Table D, Probability of positive trap according to the interaction between the location in colonized area (yes/no) and the distance to this colonized area. Trap is included as a random factor. Table E, Probability of positive trap according to the distance to colonized area and the interaction between the location in colonized area (yes/no) and the distance to this colonized area. Trap is included as a random factor. Table F, Probability of positive trap according to the type of landscape. Trap is included as a random factor. Table G, Probability of positive trap according to the current year. Trap is included as a random factor. Table H, Probability of positive trap according to the interaction between the current year and the distance to colonized area. Trap is included as a random factor. Table I, Probability of positive trap according to the mosquito presence during second semester. Trap is included as a random factor. Table J, Probability of positive trap according to the minimal temperature. Trap is included as a random factor. Table K, Probability of positive trap according to the minimal temperature, presence during second semester and their interaction. Trap is included as a random factor. Table L, Full model with trap and semester as random factor. (DOCX) [file pone.0125600.s001.docx]

**The spread of *Aedes albopictus* in Metropolitan France: contribution of environmental drivers and human activities and predictions for a near future.**

**Supplementary Materials**

Benjamin Roche^1^, Lucas Léger^2^, Grégory L’Ambert^3^, Guillaume Lacour^3,4^, Rémy Foussadier^5^, Gilles Besnard^5^, Hélène Barré-Cardi^6^, Frédéric Simard^2^, Didier Fontenille^2^

1. UMI IRD/UPMC UMMISCO, Bondy, France
2. UMR CNRS/IRD/UM1/UM2 MIVEGEC, Montpellier, France
3. EID Méditerranée, Montpellier, France
4. Centre de Recherche sur la Biodiversité, Louvain-la-Neuve, Belgique
5. EID Rhône-Alpes, Grenoble, France
6. Office de l’Environnement de la Corse, Corte, France

**A. Relationship between predicted and observed probability of *Ae. albopictus* presence.**

Here, we show the relationship between predicted and observed data. To compare the output of our model, *i.e.,* probability of *Ae. albopictus* presence, and data collected, *i.e.,* positive or negative trap, we first categorize the studied area within patches of 1km^2^ to compute the proportion of positive traps in order to compare it with probability predicted by our model within this area. Figure S1 shows that this relationship between predicted and observed values follows a relationship with a slope close to 1.

**
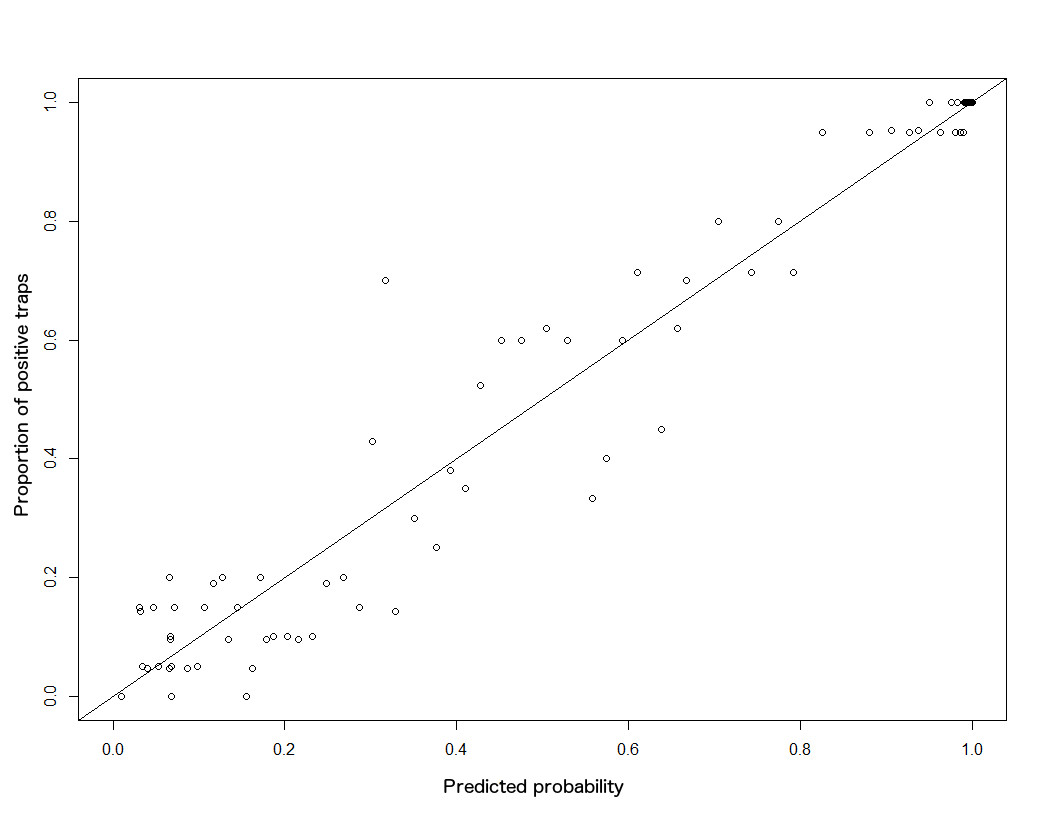
**

**Figure A: Relationship between predicted and observed data of *Ae. albopictus* presence.**

**B. Adequacy for decision-making**

Since probability of presence is not a relevant measure for public health authorities, we analyzed how this probability is distributed within positive and negative traps (figure S2). We show that considering an intuitive threshold of 50% (if the presence probability predicted is greater than 50%, the trap should be positive, otherwise it should be negative) allows to do correct classification for 95% of traps.


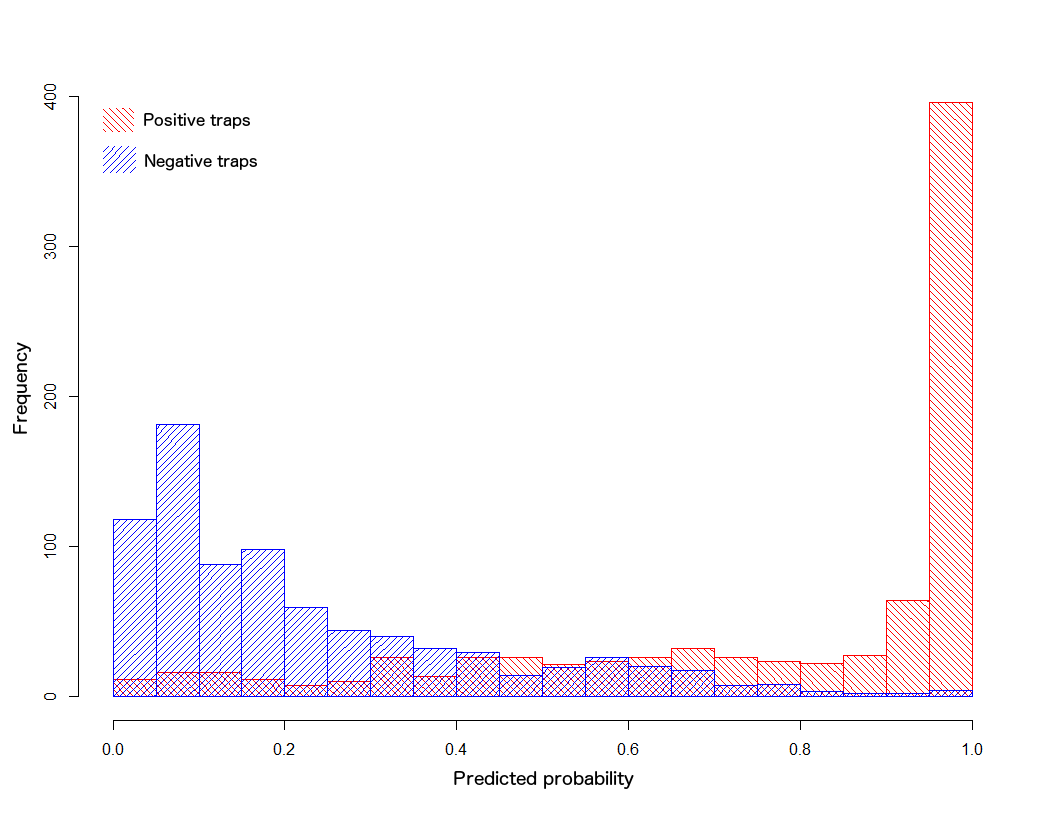


**Figure B: Distribution of *Ae. albopictus* presence probability within negative traps (in blue) and positive traps (in red).**

**C. Colinearities between variables**

The main text presents only the final model. Here, we show that variables included within this model are weakly correlated (table S1).

|  | **Distance to colonized area** | **Distance to a jump from the colonized area** | **Temperature** | **Year** |
| --- | --- | --- | --- | --- |
| **Distance to colonized area** |  | -0.088 | -0.377 | -0.286 |
| **Distance to a jump from the colonized area** | -0.088 |  | 0.116 | -0.189 |
| **Temperature** | -0.377 | 0.116 |  | -0.002 |
| **Year** | -0.286 | -0.189 | -0.002 |  |

**Table A: Correlations between variables included within the predictive model. No correlation is significant.**

**D. Models with logistic dispersion:**

In the main text, we show only models with environmental variables included for the sake of clarity. Here, we present the first step of our model, i.e., the two models explaining Ae. albopictus presence through distance from the colonized area and distance from jumps to the colonized area.

|  | Coefficients | p-value |
| --- | --- | --- |
| (intercept) | 0.9080 | 9.92e-06 |
| Distance to colonized area | -6.6316 | <2e-16 |

**Table B1: Univariate models with distance to colonized area (with a random effect ‘trap’)**

|  | Coefficients | p-value |
| --- | --- | --- |
| (intercept) | -7.2696 | <2e-16 |
| Distance to a jump from the colonized area | -3.8060 | 4.16e-06 |

**Table B2: Univariate models with distance to a jump from the colonized area (with a random effect ‘trap’).**

**E. Univariate analyses:**

In order to complete the model results presented in the main text and to highlight the model selection process we have applied, we show here all the intermediate analyses that have allowed to construct the final model:

|  | Coefficients | p-value |
| --- | --- | --- |
| (intercept) | -4.5661 | <2e-16 |
| Colonized area (yes/no) | -7.2844 | <2e-16 |

**Table C: Probability of positive trap according to location within the colonized area. Trap is included as a random factor.**

|  | Coefficients | p-value |
| --- | --- | --- |
| (intercept) | -5.7959 | <2e-16 |
| Interaction Colonized area:distance to colonized area | 30.0182 | <2e-16 |

**Table D: Probability of positive trap according to the interaction between the location in colonized area (yes/no) and the distance to this colonized area. Trap is included as a random factor.**

|  | Coefficients | p-value |
| --- | --- | --- |
| (intercept) | 0.3306 | 0.0152 |
| Distance to colonized area | -5.4892 | <2e-16 |
| Interaction Colonized area:distance to colonized area | 15/2893 | <2e-16 |

**Table E: Probability of positive trap according to the distance to colonized area and the interaction between the location in colonized area (yes/no) and the distance to this colonized area. Trap is included as a random factor.**

|  | Coefficients | p-value |
| --- | --- | --- |
| (intercept) | -9.13014 | <2e-16 |
| Land type forest | 0.07886 | 0.8959 |
| Land type peri-urban | 0.5169 | 0.3439 |
| Land type urban | 1.37 | 0.0126 |

**Table F: Probability of positive trap according to the type of landscape. Trap is included as a random factor.**

|  | Coefficients | p-value |
| --- | --- | --- |
| (intercept) | -15.3659 | <2e-16 |
| Year | 4.5316 | <2e-16 |

**Table G: Probability of positive trap according to the current year. Trap is included as a random factor.**

|  | Coefficients | p-value |
| --- | --- | --- |
| (intercept) | -9.6554 | <2e-16 |
| Interaction Year:Distance to colonized area | 1.1013 | <2e-16 |

**Table H: Probability of positive trap according to the interaction between the current year and the distance to colonized area. Trap is included as a random factor.**

|  | Coefficients | p-value |
| --- | --- | --- |
| (intercept) | -11.3115 | <2e-16 |
| Second semester | 1.47008 | <2e-16 |

**Table I: Probability of positive trap according to the mosquito presence during second semester. Trap is included as a random factor.**

|  | Coefficients | p-value |
| --- | --- | --- |
| (intercept) | -7.9697 | <2e-16 |
| Minimal temperature | 0.1044 | <2e-16 |

**Table J: Probability of positive trap according to the minimal temperature. Trap is included as a random factor.**

|  | Coefficients | p-value |
| --- | --- | --- |
| (intercept) | -11.7706 | <2e-16 |
| Second semester | 1.636 | <2e-16 |
| Minimal temperature | 0.117 | <2e-16 |
| Interaction Second semester: Minimal temperature | -0.01212 | <2e-3 |

**Table K: Probability of positive trap according to the minimal temperature, presence during second semester and their interaction. Trap is included as a random factor.**

**F. Other ways to add random intercept:**

To take into account temporal autocorrelation, we have also tried to include year in addition to trap as a random variable. This model (shown below) is not significantly better than the model presented in the main text.

| Variable |  | Coefficient |
| --- | --- | --- |
| Agricultural landscape |  | 0.826 |
| Peri-urban landscape |  | 0.7536 |
| Urban landscape |  | 1.1944 |
| Second semester |  | 1.4294 |
| Minimum temperature of the coldest month |  | 0.0527 |
| Distance to colonized area*colonized area |  | 12.6971 |
| Second semester*minimum temperature |  | -0.0124 |
| Distance to colonized area*year |  | 3.0013 |
| Distance to colonized area at previous semester |  | -9.563 |
| Distance to area sporadically colonized |  | -6.484 |
| Area Under the Curve (AUC): |  | Fixed effect: 0,96  Fixed and random effect: 0,99  R2c=0.88 |

**Table L: Full model with trap and semester as random factor.**
